# Genotoxic *Klebsiella pneumoniae* in Taiwan

**DOI:** 10.1371/journal.pone.0096292

**Published:** 2014-05-22

**Authors:** Yi-Chyi Lai, Ann-Chi Lin, Ming-Ko Chiang, Yu-Han Dai, Chih-Chieh Hsu, Min-Chi Lu, Chun-Yi Liau, Ying-Tsong Chen

**Affiliations:** 1 Department of Microbiology and Immunology, Chung-Shan Medical University, Taichung City, Taiwan; 2 Division of Infectious Diseases, Department of Internal Medicine, Chung-Shan Medical University Hospital, Taichung City, Taiwan; 3 Institute of Microbiology and Immunology, Chung-Shan Medical University, Taichung City, Taiwan; 4 Institute of Molecular and Genomic Medicine, National Health Research Institutes, Miaoli County, Taiwan; 5 Department of Life Science, National Chung Cheng University, Chia-Yi County, Taiwan; 6 Institute of Genomics and Bioinformatics, National Chung Hsing University, Taichung City, Taiwan; 7 Biotechnology Center, National Chung Hsing University, Taichung City, Taiwan; Peking University Cancer Hospital and Institute, China

## Abstract

**Background:**

Colibactin is a nonribosomal peptide-polyketide synthesized by multi-enzyme complexes encoded by the *pks* gene cluster. Colibactin-producing *Escherichia coli* have been demonstrated to induce host DNA damage and promote colorectal cancer (CRC) development. In Taiwan, the occurrence of pyogenic liver abscess (PLA) has been suggested to correlate with an increasing risk of CRC, and *Klebsiella pneumoniae* is the predominant PLA pathogen in Taiwan

**Methodology/Principal Findings:**

At the *asn* tRNA loci of the newly sequenced *K. pneumoniae* 1084 genome, we identified a 208-kb genomic island, KPHPI208, of which a module identical to the *E. coli pks* colibactin gene cluster was recognized. KPHPI208 consists of eight modules, including the colibactin module and the modules predicted to be involved in integration, conjugation, yersiniabactin production, microcin production, and unknown functions. Transient infection of BALB/c normal liver cells with *K. pneumoniae* 1084 increased the phosphorylation of histone H2AX, indicating the induction of host DNA damage. Colibactin was required for the genotoxicity of *K. pneumoniae* 1084, as it was diminished by deletion of *clbA* gene and restored to the wild type level by trans-complementation with a *clbA* coding plasmid. Besides, BALB/c mice infected with *K. pneumoniae* 1084 exhibited enhanced DNA damage in the liver parenchymal cells when compared to the isogenic *clbA* deletion mutant. By PCR detection, the prevalence of *pks*-positive *K. pneumoniae* in Taiwan is 25.6%, which is higher than that reported in Europe (3.5%), and is significantly correlated with K1 type, which predominantly accounted for PLA in Taiwan.

**Conclusions:**

Our knowledge regarding how bacteria contribute to carcinogenesis has just begun. The identification of genotoxic *K. pneumoniae* and its genetic components will facilitate future studies to elucidate the molecular basis underlying the link between *K. pneumoniae*, PLA, and CRC.

## Introduction

*Klebsiella pneumoniae* is a member of the family *Enterobacteriaceae*. The ability of *K. pneumoniae* to cause a wide range of human diseases, from urinary tract infections to life-threatening systemic infections, has attracted increasing attention to the pathogenesis of this bacterium [1]–[4]. In 1990s, *K. pneumoniae* surpassed *Escherichia coli* as the leading cause of community-acquired pyogenic liver abscess [5]. *K. pneumoniae*-caused liver abscess (KLA) was recognized by researchers in Taiwan and several other areas since 1986. KLA is now considered an emerging disease worldwide [1]–[3], [6]. Distinct from *E. coli*-caused liver abscess, KLA is generically cryptogenic and is frequently complicated with metastatic lesions to other organs [7]–[9]. A significant proportion of KLA isolates in Taiwan belong to capsular serotype K1, with a prevalence rate ranging from 28% to 63% [10], [11]. Several K1-specific genetic loci have been identified to contribute to *K. pneumoniae* virulence, including *magA* (for capsular polysaccharide synthesis), *kfu*/PTS (for iron uptake), and genes for the TonB-dependent iron acquisition system (*iucABCDiutA*, *iroAiroNDCB*, and yersiniabactin island) [12]–[15].

Colorectal cancer (CRC) is a significant cause of morbidity and mortality throughout the world. Most CRC occur in people without a family history. Age, history of inflammatory bowel disease, and lifestyle are known risk factors for CRC. In Taiwan, CRC is the second most commonly diagnosed cancer. Its annual incidence increases steadily from 34.43/100,000 in year 2000 to 54.01/100,000 in year 2009 according to the report from the Department of Health in Taiwan. Pyogenic liver abscess has been suggested as a potential manifestation of CRC in Taiwan [16]–[19]. Intriguingly, an 11-year follow-up study in Taiwan demonstrated that patients with *K. pneumoniae*-caused PLA (KLA) were at increased risk of subsequent CRC than did patients with non-*K. pneumoniae* PLA [20]. This finding raises a possibility that some bacterial virulence factors may endow *K. pneumoniae* with a cancer-inducing capacity.

Since the identification of an oncogenic role for *Helicobacter pylori* [21], our knowledge regarding how bacteria contribute to tumorigenesis has just begun. Acquisition of genomic instability is crucial for initiation and progression of tumors. Chronic exposure to DNA damaging agents may cause elevated genomic instability as a result of failure to repair damaged DNA or altered activation of the DNA damage-induced checkpoint responses [22]. Two bacterial products, cytolethal distending toxin (CDT) [23] and colibactin [24] have been identified as genotoxins that can directly damage DNA, inhibit cell cycle, and trigger genomic instability in the host [22], [24]. CDT is a tripartite exotoxin consisting of CdtA, CdtB, and CdtC encoded by the *cdtABC* operon. Numerous bacteria have been found to produce CDT, including *E. coli*, *H. pylori*, *Haemophilus ducreyi*, *Shigella dysenteriae*, *Salmonella enterica*, and *Campylobacter spp.* [23], [25]. Colibactin, on the other hand, is a hybrid nonribosomal peptide-polyketide [24], [26] synthesized by multi-enzyme complexes that are encoded by genes carried on a 54-kb genomic region of *E. coli* [24]. The 54-kb chromosomal region, the *pks* colibactin gene cluster, was identified and fully sequenced in newborn meningitis *E. coli* strain IHE3034 [24]. In *E. coli*, the genotoxicity conferred by the *pks* colibactin gene cluster were demonstrated in vitro and in vivo [22], [24], [26]. By PCR, the presence of colibactin genes was detected in 53% of extraintestinal pathogenic *E. coli* (ExPEC) strains and in 34% of fecal *E. coli* isolates from healthy individuals [24]. The colibactin-positive *E. coli* isolates are almost exclusively classified in the phylogenic lineage ECOR-B2 [24]. In addition to *E. coli*, few members of the family *Enterobacteriaceae* were found positive on the carriage of *pks* colibactin gene cluster, including *Enterobacter aerogenes*, *Citrobacter koseri*, and *K. pneumoniae* [27].

Horizontal gene transfer contributes to the evolution and emergence of pathogens by allowing virulence-associated genes to spread among bacteria [28]. Clusters of genes can be acquired as a unit known as genomic island. Several genomic islands have been discovered in *K. pneumoniae*, including the high pathogenicity genomic island HPI-ICE*Kp1* at the *asn* tRNA loci of *K. pneumoniae* NTUH K2044 [29], fimbrial gene cluster-containing KPGI-5 at *met* tRNA loci of *K. pneumoniae* KR116 genome [30], and several genomic islands related to carbohydrate metabolism and toxin synthesis [31], [32]. Recently, we determined the complete genome sequence of *K. pneumoniae* 1084 (GenBank Accession No. NC_018522.1) [33], which was a K1 strain isolated in Taiwan from a KLA patient. A comparative genomics approach was performed to identify potential determinants of virulence in the newly sequenced genome. Here we report the identification of a 208-kb chromosomal region with typical features of a genomic island at the *asn* tRNA loci of the *K. pneumoniae* 1084 genome. This 208-kb genomic island, named KPHPI208, is composed of 8 genomic modules (GMs). GM1, the first genomic module of KPHPI208, contains genes ∼100% identical to those of the *pks* colibactin gene cluster reported in *E. coli* IHE3034 [24]. The colibactin-related genotoxicity of *K. pneumoniae* 1084 was validated by *in vitro* and *in vivo* experiments. The prevalence of colibactin genes among clinical *K. pneumoniae* isolates in Taiwan was also investigated and reported herein.

## Materials and Methods

### Ethics statement

Bacterial strains were isolated from patients with primary *K. pneumoniae* infections at Chung-Shan Medical University Hospital in central Taiwan during a 15-month period from April 2002. The isolates were cultured from clinical samples with no collection of patient identifiers or interaction with subjects. All animal experiments were performed in strict accordance with the recommendation in the Guide for the Care and Use of Laboratory Animals of the National Laboratory Animal Center (Taiwan), and the protocol was approved by the Animal Experimental Center of Chung-Shan Medical University.

### Bacterial strains

A total of 207 non-repetitive *K. pneumoniae* isolates were collected in our previous study [11]. Among them, 35 (16.9%) were regarded as KLA strains as they were obtained from tissue-invasive cases that presented with the formation of liver abscesses, 59 (28.5%) were from cases associated with abscesses at non-hepatic sites, including lesions that occurred as empyema, endophthalmitis, necrotizing fasciitis, septic arthritis, along with lung, epidural, parotid, paraspinal, splenic, renal, prostate, muscle, and deep neck abscesses, and the remaining 113 (54.6%) isolates were obtained from non-abscess-related cases, including pneumonia without abscess, primary peritonitis, cellulitis, biliary tract infection, primary bacteremia with no original infectious foci identifiable, and catheter-related infections. The *K. pneumoniae* isolates were cultured in Luria-Bertani (LB) broth and stored at −80°C until use. Capsular antigens were determined by PCR detection of the K-serotype-specific *wzx* locus. Specific primers designed previously [7] were used to detect K1 type (GTA GGT ATT GCA AGC CAT GC and GCC CAG GTT AAT GAA TCC GT), and K2 type (GGA GCC ATT TGA ATT CGG TG and TCC CTA GCA CTG GCT TAA GT). *K. pneumoniae* 1084 is a K1 strain isolated from a diabetic patient with severe bacteremic liver abscess. Complete sequencing and annotation of *K. pneumoniae* 1084 was performed in our previous study (GenBank Accession No. NC_018522.1) [33].

### Deletion and complementation of *clbA*

A 768-bp region spanning the coding sequence of *clbA* was deleted in *K. pneumoniae* 1084 by using an allelic exchange technique as described [34]. In general, ∼1,200-bp DNA fragments flanking the region to be deleted were amplified with specific primer sets, p465 (AAA ATC TAG ACA TAG AGT TGG AGC AAC TGT T)/p466 (AAA AGG TAC CCT CAT TCC TGT TAG CAA TGT G) and p467 (AAA AGG TAC CTC TGA GCC GTC GAT AAT ATT GA)/p468 (AAA AGA GCT CTC CTA CCC TCG TAA TAT GGA CA) and the amplified DNA fragments were cloned into pKAS46, a suicide vector containing *rpsL*, which allows positive selection for vector loss using streptomycin [35]. After the occurrence of double crossover, the streptomycin-resistant but kanamycin-sensitive colonies were selected. The deletion of *clbA* was verified by PCR and Southern blot analysis and one of the confirmed mutants was named Δ*clbA*. For complementation experiments, a 735-bp DNA fragment containing full-length *clbA* gene was amplified using primers p478 (ATG AGG ATT GAT ATA TTA ATT GGA C) and p479 (ATT CTG CCC ATT TGA CGA ATG). The amplified DNA fragment was cloned into pCR®II (Invitrogen) to generate pYC502. Complementation of *clbA* was performed by introducing plasmid pYC502 into Δ*clbA* via electroporation.

### *In vitro* infection assay

BALB/c normal liver (BNL) cells (ATCC TIB-73) were maintained by serial passage in Dulbecco modified Eagle medium (DMEM) supplemented with 5% fetal bovine serum at 37°C with 5% CO_2_. The BNL cells were synchronized by switched to serum free medium for 16 hours. Approximately at 70% confluence, the BNL cells were washed four times with phosphate-buffered saline (PBS) and infected with *K. pneumoniae* 1084, Δ*clbA*, or Δ*clbA*-pYC502 at a multiplicity of infection (MOI) of 100 bacteria per cell. After a 4-h transient infection, BNL cells were washed with PBS and cultured with fresh DMEM supplemented with 5% fetal bovine serum (FBS) and 100 µg/ml gentamycin. For clonogenic assay, the control and infected cells were trypsinized to produce a single cell suspension. Equal amount of cells (1.5×10^2^) were seeded into a 35 mm culture dish. After incubation for 14 days, cells were fixed then stained with 0.5% crystal violet. The number of colonies for each group was counted under stereomicroscope in a double-blind manner. To detect phospho-H2AX (γH2AX) foci in the nuclei, cells were fixed with 95% methanol-5% acetic acid, blocked with PBS-0.1% Tween 20–2% skim milk for 30 min, probed with rabbit anti-phospho-H2AX antibody (Cell Signaling Technology), and then hybridized with goat anti-rabbit-Alexa488 antibodies (Invitrogen). A Zeiss LSM510 CLSM (Carl Zeiss) with a 60× oil objective was used for imaging. At least 300 cells per group were counted for phospho-H2AX staining from images in a double-blind manner. For Western blot, ∼10^7^ cells were collected and lysed with 1000 µl of lysis buffer (PBS, 1% Triton X-100, with protease and phosphatase inhibitors). After denaturation by boiling for 5 min with loading buffer, 30 µg of total proteins were separated on 12% of SDS gel, transferred to PVDF membrane (BioRad), blocked with 2% skim milk, and probed with anti-phospho-H2AX (Cell Signaling Technology), or with anti-Histone H3 (Cell Signaling Technology) antibodies, followed by horseradish peroxidase-conjugated secondary antibodies, and chemiluminescence detection.

### *In vivo* infection assay

Male BALB/c mice were purchased from the National laboratory animal center (NLAC Taiwan) at 7 wk of age and allowed to acclimatize in the animal house of Chung Shan Medical University for 1–2 wk before experiments. Mice were starved for 16 h prior to infection. PBS or 100 µl of bacterial suspension containing 10^9^ CFU of *K. pneumoniae* 1084, Δ*clbA*, or Δ*clbA*–pYC502 was respectively inoculated into groups of mice (6 mice a group) via an oral route as described in our previous study [34]. The PBS-inoculated or *K. pneumoniae*-infected mice were sacrificed at 1 day or 2 days post-inoculation. The liver was recovered immediately. The retrieved liver was divided into two parts, one was homogenized for determination of bacterial loads and detection of the level of phospho-H2AX by Western blot analyses, and the other was fixed in 4% paraformaldehyde at 4°C for 24 hours then embedded in paraffin. After deparaffinization and rehydration, slides were prepared and incubated with boiling sodium citrate buffer (pH 6.0; 10 mM) for 10 min to unmask antigens, and stained with rabbit anti-phospho-H2AX (Thermo Fisher Scientific) antibodies. Signals were detected by using a NovoLink Polymer Detection System (Leica Biosystem). Counterstaining was done with nuclear Hematoxylin. Images were acquired with a Nikon light microscope. Three sections were randomly selected from each of the samples retrieved from experimental mice in a double-blind manner. At least 1000 hepatocytes per section were counted for phospho-H2AX-positive staining.

### PCR detection of *pks* colibactin genes

The presence of *pks* colibactin genes among clinical *K. pneumoniae* isolated in Taiwan was determined by PCR with primers as published in a previous study [24]. Briefly, primers for *clbB* and *clbN* were clbBF (GAT TTG GAT ACT GGC GAT AAC CG), clbBR (CCA TTT CCC GTT TGA GCA CAC), clbNF (GTT TTG CTC GCC AGA TAG TCA TTC), and clbNR (CAG TTC GGG TAT GTG TGG AAG G). Primers for two internal loci within the *pks* colibactin gene cluster, *clbA* (phosphopantetheinyl transferase) and *clbQ* (thioesterase), were clbAF (CTA GAT TAT CCG TGG CGA TTC), clbAR (CAG ATA CAC AGA TAC CAT TCA), clbQF (CTT GTA TAG TTA CAC AAC TAT TTC), and clbQR (TTA TCC TGT TAG CTT TCG TTC). Genomic DNA (gDNA) samples for the 207 *K. pneumoniae* strains were prepared as described [11]. An initial denaturation at 95°C for 10 min was followed by denaturation at 94°C for 45 s, annealing at 54°C for 45 s, and extension at 72°C for 1 min for 30 cycles. *K. pneumoniae* 1084 was used as the positive control and *K. pneumoniae* CG43 was used as the negative control for the PCR experiments.

### Statistical analysis

The Mann-Whitney U test, Student's *t* test, or Fisher's exact test were used to determine differences between groups suggested. Statistical significance was determined based on a two-tailed *P* value <0.05.

## Results and Discussion

### Genetic structure and comparative analysis of KPHPI208

**Figure 1:**
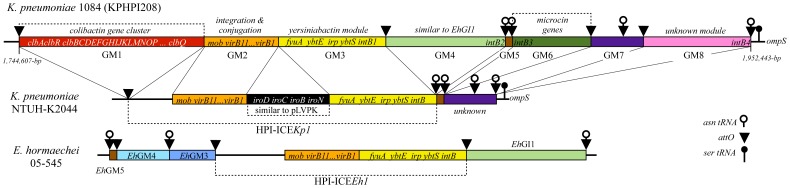
Genetic structure of KPHPI208 and related genomic islands. Modules in the genomic regions were depicted as colored boxes and the important genes were labeled (GM1∼8). Red: the *pks* colibactin genes; orange: the genes responsible for integration and conjugation of the ICE element; yellow: the yersiniabactin genes; light green: unknown function, similar to genomic module *Eh*GI1 from ICE*Eh1* (GenBank accession No: FN297818); brown: unknown function, similar to *Eh*GM5 of ICE*Eh1*; dark green: microcin E492 genes, similar to the microcin genomic island in *K. pneumoniae* RYC492 (GenBank accession No: AF063590); purple and pink: unknown. The *attO* sequences were marked by triangles. The positions of the tRNA genes were also marked along the region.

At the *asn* tRNA loci, an integration hotspot for foreign mobile DNA elements, we identified a 208-kb region exhibiting typical features of a genomic island and named it KPHPI208. A total of 135 protein-coding genes were contained in this island. Its overall G+C% is 49.6, which is lower than that of the entire genome of *K. pneumoniae* 1084 (57.4). Comparative genomics analysis of this region had identified eight genomic modules (GM1∼GM8) each displaying a conserved genetic organization and were likely exchangeable among bacteria genomes. As shown in Figure 1, KPHPI208 accommodates a *pks* colibactin module (GM1), 5 modules (GM2, GM3, GM4, GM5, and GM7) similar to those contained within HPI-ICE*Kp1* [29] and HPI-ICE*Eh1* [36], a microcin module (GM6), and a novel module (GM8). The insertion site of KPHPI208 is located at the *asn* tRNA locus near the *ompS* gene of the *K. pneumoniae* 1084 genome. Homologous regions of GM2, GM3, GM4, and GM5 were identified in the genome of *Enterobacter hormaechei* 05-545 spanning the high pathogenicity island HPI-ICE*Eh1* [36], and in the genome of *E. coli* ECOR31 at the integrative and conjugative element ICE*Ec1* [37]. GM2 is an 18-kb module containing genes for integration and conjugation. GM3 is a 30-kb module carrying genes for yersiniabactin production. GM4 and GM5 harbor genes of unknown functions (Figure 1). Homologues of GM2, GM3, GM5, and GM7 also present in the 76-kb high pathogenicity island HPI-ICE*Kp1* of *K. pneumoniae* NTUH K2044 [29]. The insertion site of HPI-ICE*Kp1* in the genome of NTHU K2044 is also the *asn* tRNA locus near *ompS*. Nucleotide sequence of GM6 is ∼100% identical to the microcin E492 gene cluster of *K. pneumoniae* RYC492 [32]. The 22-kb region consisting of genes for the production of microcin and immunity protein locates next to the second *asn* tRNA locus in KPHPI208. The eight modules (GM1-GM8) of KPHPI208 are bounded by 4 *asn* tRNA genes, 8 *attO* sites, and 4 *intB* integrase genes (Figure 1). The *attO* is a conserved 17-bp direct repeat that served as an integration site for integrative and conjugative elements (ICEs). In compared to HPI-ICE*Kp1*, which has only one integrase gene (*intB1*) in the yersiniabactin module, KPHPI208 has three additional *intB* genes, *intB2*, *intB3*, and *intB4*, carried by GM4, GM6, and GM8, respectively (Figure 1). Acquirement of the three modules flanked by *attO* and *asn* tRNA was probably the result of insertion events occurred at the three *asn* tRNA loci of the *K. pneumoniae* genomes (Figure 1).

**Figure 2:**
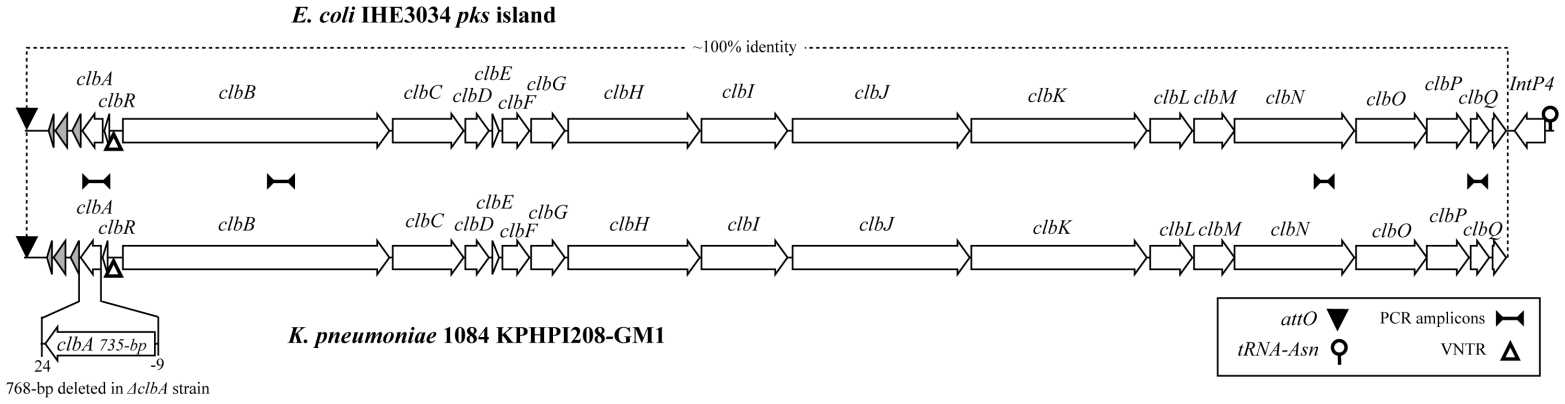
The *pks* colibactin gene cluster (GM1) in KPHPI208 and the *pks* colibactin gene cluster in the *E. coli* IHE3034 genome. The regions spanning the genes responsible for colibactin production were depicted as arrows according to the directions of transcription. The *attO* sites in the left were marked by solid triangles. The VNTR locus between *clbR* and *clbB* was marked by empty triangles. The 53-kb regions indicated by dotted lines are ∼100% identical. The locations of the four PCR amplicons in studying the prevalence of the colibactin genes among *K. pneumoniae* clinical isolates were marked. The 768-bp region spanning the *clbA* gene, which was deleted in *ΔclbA* strain, was indicated.

GM1, the largest module in KPHPI208, consists of genes responsible for colibactin production. Nucleotide sequence of GM1 is ∼100% identical to that of the *pks* colibactin gene cluster reported in *E. coli* IHE3034 [24] (Figure 2). Colibactin is a peptide-polyketide hybrid compound. For the synthesis of colibactin, the *pks* colibactin gene cluster encodes several proteins including non-ribosomal peptide megasynthases (NRPS), polyketide megasynthases (PKS), hybrid NPRS/PKS megasynthases, and accessory, tailoring, and editing enzymes [24]. GM1 of KPHPI208 accommodates all of the *pks* genes required for the synthesis of colibactin (Figure 2). In the genome of *E. coli* IHE3034, the *pks* colibactin gene cluster is flanked by *attO* direct repeats at both sides, and an additional P4-like integrase gene, *intP4*, is located next to the end of the gene cluster near *clbQ* [27]. Although the *pks* colibactin gene cluster carried in KPHPI208-GM1 is identical to the *E. coli* version, the *intP4*, together with one of the *attO* next to it, are not found. This is reminiscent of a previous study, in which five out of 141 *K. pneumoniae* isolates from Europe were PCR-detected positive on the presence of colibactin genes. In the study the *intP4* associated with the colibactin gene cluster was reported to be missing in *K. pneumoniae* [27]. Sequencing of KPHPI208 not only confirmed this genetic structure but also uncovered the complete genetic structure of the genomic island by discovering additional genomic modules in this region. In addition, the variable-number tandem repeat (VNTR) between *clbB* and *clbR* of KPHPI208-GM1 has 16 ACAGATAC repeats. It is 13 repeats in *E. coli* IHE3034 (Figure 2).

### Genotoxicity of *K. pneumoniae* 1084

**Figure 3:**
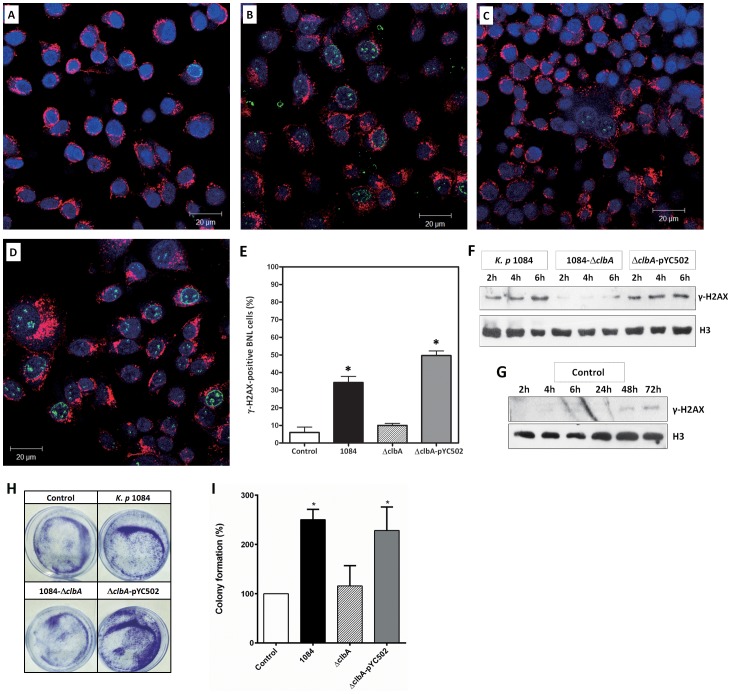
*K. pneumoniae* 1084 induced colibactin-related DSBs *in vitro*. BNL cells were left uninfected (A) or were infected with *K. pneumoniae* 1084 (B), Δ*clbA* (C), or with Δ*clbA* complemented with *clbA* coding plasmid pYC502 (D) at an MOI of 100. After 4 h infection, the cells were washed, co-cultured with gentamycin (100 µg/ml), and were examined by confocal microscopy for DNA (blue, stained with Hoechst 33342), for membrane glycoproteins (red, stained with ConA), and for γH2AX (green, recognized by Alexa488-anti-γH2AX antibodies) (scale bar, 20 µm). (E) Quantification of γH2AX-positive cells. Error bars represent SEs from three experiments. (F) Western blot analyses of γH2AX or H3 in BNL cells recovered at 2, 4, and 6 h after a 4 h transient infection with *K. pneumoniae* 1084 (lanes 1–3), Δ*clbA* (lanes 4–6), or with Δ*clbA* complemented with *clbA*-coding plasmid pYC502 (lanes 7–9). (G) Western blot analyses of γH2AX and H3 in uninfected BNL cells harvested from serum recovery for 2, 4, 6, 24, 48, and 72 h. (H) Clonogenic assays. BNL cells were uninfected (Control) or transiently infected with *K. pneumoniae* 1084, Δ*clbA*, or with Δ*clbA*-pYC502 for 4 hours. Colonies formed after 14-day incubation stained with 0.5% of crystal violet. A representative image is presented. (I) Quantification of colony formation. Error bars represents SEMs from three experiments. An asterisk (*) represents a significant increase in the *K. pneumoniae*-infected group in comparison with the uninfected control by the Student's *t* test (two-tailed; *P*<0.05).

Exposure to colibactin-producing *E. coli* was demonstrated to inflict double strand breaks (DSBs) of host DNA in vitro and in vivo [22], [24], [26]. Discovery of the *pks* colibactin gene cluster in KPHPI208-GM1 (Figure 2) encouraged us to examine whether this gene cluster endowed *K. pneumoniae* 1084 with genotoxicity. DSBs of host DNA induce phosphorylation of the histone protein H2A variant (H2AX) at serine 139 that generates γ-H2AX through the activation of ATM (Ataxia telangiectasia mutated) and ATR (Ataxia telangiectasia and Rad3-related protein) kinases. Accumulation and retention of γ-H2AX can therefore serve as an indicator for the signaling cascade induced by DNA damage [38]. In this study, BALB/c normal liver (BNL) cells were used as a cell model to examine the degree of DNA damage induced by *K. pneumoniae* 1084 infection. Compared to the uninfected control (Figure 3A), a number of γH2AX foci were noted in the nuclei of *K. pneumoniae* 1084-infected BNL cells at 4 h post-infection (green fluorescent signals in Figure 3B). Because the γH2AX foci on mitotic chromosomes represent unrepaired DNA breaks or scar of repaired lesions [39], [40], this result suggests an induction of DNA damage by *K. pneumoniae* 1084 infection. A phosphopantetheinyl transferase is encoded by *clbA* that is essential for the synthesis of colibactin [24]. It was demonstrated in *E. coli* that the genotoxicity in host cells was significantly attenuated by the loss of *clbA* [22], [26]. To verify whether the *K. pneumoniae* 1084-induced DNA damage was attributed by colibactin, we deleted a 768-bp region spanning the coding sequence of *clbA* (Figure 2) in *K. pneumoniae* 1084 to generate an isogenic *clbA* mutant and named it Δ*clbA*. Similarly, the γH2AX foci were hardly detected in the *K. pneumoniae* Δ*clbA*-infected BNL cells (Figure 3C). By Western blot analyses, time-dependent accumulation of γH2AX was noted in the transiently infected BNL cells, which were recovered at 2, 4, and 6 h after a 4-h infection with *K. pneumoniae* 1084 (Figure 3F), as compared with the uninfected control (Figure 3G). The γH2AX signal was diminished in the Δ*clbA*-infected group (Figure 3F). By introduction of the *clbA*-complementing plasmid pYC502, the reduced genotoxicity of Δ*clbA* was restored to the wild type level (Figure 3D, 3E and 3F). The result demonstrated the genotoxicity of *K. pneumoniae* 1084, which was conferred by the colibactin genes accommodated in KPHPI208-GM1. Viability of the *K. pneumoniae* 1084-infected cells was unaffected upon a 4-h transient infection. To demonstrate the consequence of the DNA damage induced on BNL cells by infection with *K. pneumoniae* 1084, we performed a conventional clonogenic assay. After 14-day incubation, the number of colonies formed in the *K. pneumoniae* 1084- and the complement strain (Δ*clbA*-pYC502)-infected group was significantly greater than that in the Δ*clbA*-infected group (Figure 3H and 3I). This result suggested that the accumulation of *K. pneumoniae* 1084-induced DNA damage might contribute to tumorigenesis.

**Figure 4:**
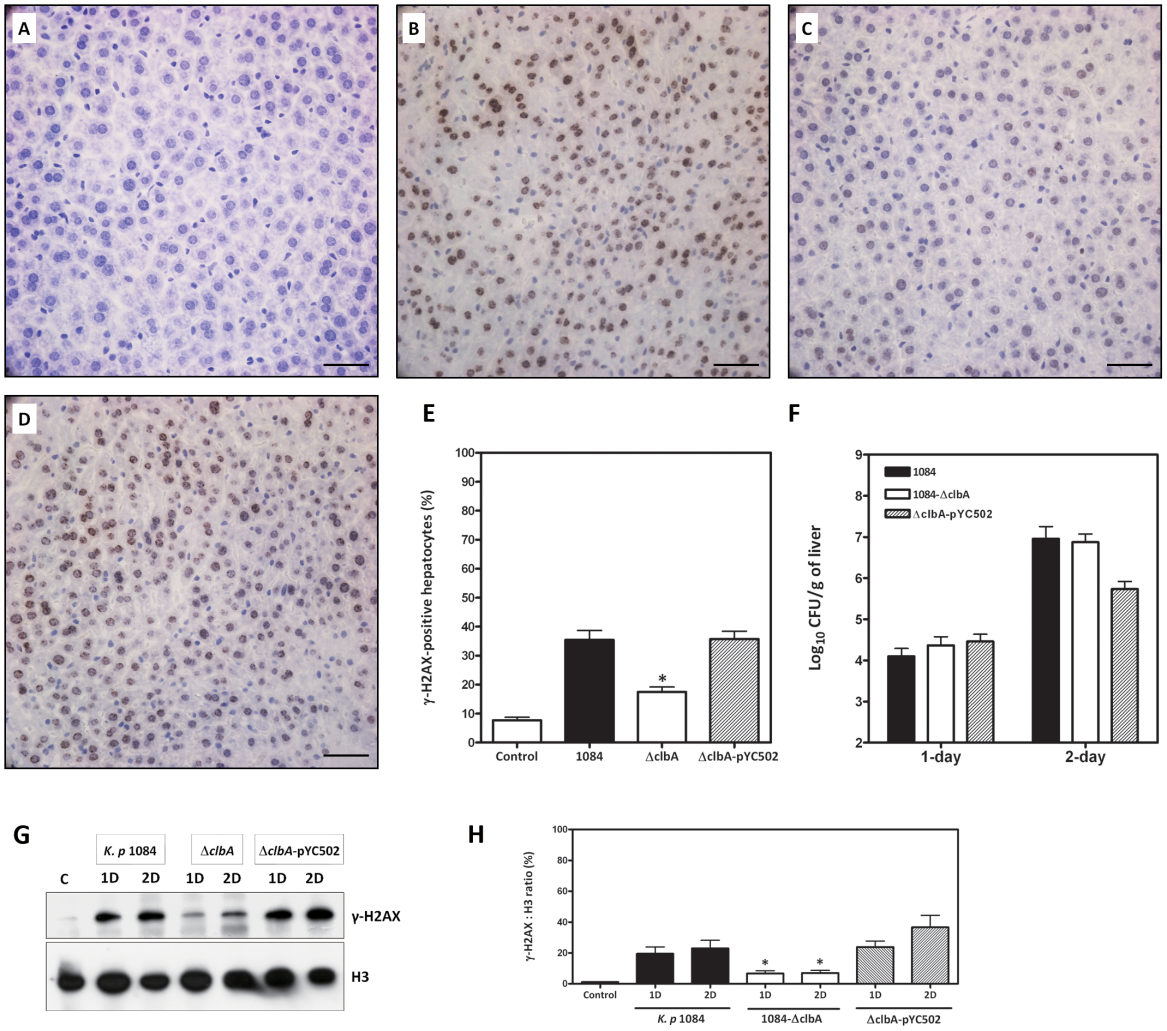
DNA damage evoked by *K. pneumoniae* 1084 infection *in vivo*. For all the experimental groups, liver sections were prepared from the liver retrieved at 2-day post-inoculation, stained with anti-γH2AX antibodies, and imaged under microscopic observation with magnification of 400×. Representative liver section of the PBS-inoculated control mice (A), the *K. pneumoniae* 1084-infected mice (B), the Δ*clbA*-infected mice (C), and the Δ*clbA*-pYC502-infected mice (D) are shown. Scale bar: 50 µm. (E) Quantification of γH2AX-positive hepatocytes. Each value is the mean ± SEM for 3 mice. An asterisk (*) represents a significant decrease in the Δ*clbA*-infected group in comparison with the *K. pneumoniae* 1084-infected group by the Mann-Whitney U test (two-tailed; *P*<0.05). (F) Bacterial loads of the liver determined at 1-day and 2-day infection with *K. pneumoniae* 1084 (black bars), Δ*clbA* (white bars), or Δ*clbA*-pYC502 (slash bars). Ten-folded dilutes of liver homogenates were plated onto M9 agar for enumerating CFU of *K. pneumoniae*. Each value is presented as the average bacterial loads ± SEM (Log CFU/g) for 3 mice a time point. Statistical analysis by the Mann-Whitney U test showed no significant difference between the *K. pneumoniae* 1084-infected and the Δ*clbA*-infected groups. (G) Liver lysates were prepared from PBS-control, *K. pneumoniae* 1084-, Δ*clbA*-, and Δ*clbA*-pYC502-infected mice at 1-day and 2-day post-inoculation. The sample size in each experimental group for each time point is 3. Eighty micrograms of total proteins were subjected to Western blot analyses with specific antibodies against γH2AX and H3. A representative result from at least three experiments is shown. (H) Band intensity of γH2AX and H3 was determined by Densitometry calculation and the average ratio of γH2AX to H3 is presented. Each value is the mean ± SEM for 3 mice. An asterisk (*) represents a significant decrease in the Δ*clbA*-infected group in comparison with the *K. pneumoniae* 1084-infected group by the Mann-Whitney U test (two-tailed; *P*<0.05).

To further determine whether *K. pneumoniae* 1084 elicited DNA damage *in vivo*, 10^9^ CFU of *K. pneumoniae* 1084 were orally inoculated into 8–10 week old BALB/c mice. At 1-day or 2-day post-inoculation, mice were sacrificed and the liver was retrieved for tissue section. Compared with the PBS-inoculated control mice (Figure 4A), extensive distribution of γH2AX foci were observed in the nuclei of liver parenchymal cells infected with *K. pneumoniae* 1084 (Figure 4B) or with the complement strain (Δ*clbA*-pYC502) (Figure 4D). The number and intensity of γH2AX foci in the *K. pneumoniae* Δ*clbA*-infected liver was significantly reduced at 1-day and 2-day post-inoculation (Figure 4C and 4E, *P*<0.05). Although the loss of *clbA* significantly attenuated the genotoxicity of *K. pneumoniae* 1084, it did not affect the ability to disseminate into liver from the intestines. Bacterial loads in livers retrieved at 1-day and 2-day post-inoculation were comparable among the *K. pneumoniae* 1084-, Δ*clbA*-, and Δ*clbA*-pYC502-infected groups (Figure 4F). The decreased level of γH2AX in the Δ*clbA*-infected livers was confirmed by Western blot analyses (Figure 4G and 4H). Our results suggested that the genotoxicity may not contribute to the dissemination of *K. pneumoniae* into extra-intestinal tissues. It is not surprising that *pks* island is absent in hyper-virulent *K. pneumoniae* isolates from KLA patients, such as CG43 or NTUH-K2044 [12]. Collectively, similar to *E. coli* IHE3034 [22], [24], [26], as a carrier of the *pks* colibactin gene cluster, *K. pneumoniae* 1084 displayed a genotoxic phenotype that induces DNA damage *in vivo* and *in vitro*.

As demonstrated by *Cuevas-Ramos et al* [22], DNA damage elicited by a short exposure to *E. coli* IHE3034 could trigger genomic instabilities. Because cell division cycles are resumed rapidly after a transient DNA damage response, in the dividing cells, accumulation of DNA breaks and propagation of breakage-fusion-bridge cycles can consequently evoke persistent chromosome aberrations, such as micronuclei, aneuploidy, ring chromosome, and anaphase bridges. If cells harboring chromosome aberrations continue to proliferate in the presence of DNA damage, the gene mutation frequency and transformed phenotype would be enhanced [22]. Given the nucleotide sequence and genetic structure of the *pks* colibactin gene cluster accommodated in KPHPI208-GM1 of *K. pneumoniae* 1084 is identical to the *E. coli* IHE3034 version [24] (Figure 2), we speculate that by accumulating DNA damage elicited by *K. pneumoniae* 1084 infection, genomic instability of host cells could also be triggered. Therefore, tumorigenesis might be promoted by colonization and/or infection with *K. pneumoniae* that carries the *pks* colibactin gene cluster by inducing DNA damage and the subsequent genomic instabilities in host cells.

### Prevalence of the colibactin genes among clinical *K. pneumoniae* isolates in Taiwan

The presence of *pks* colibactin gene cluster as a part of the mobilome suggests its potential to spread among local *K. pneumoniae* strains. To investigate the prevalence of colibactin genes (Figure 2) among *K. pneumoniae* isolates in Taiwan, PCR detection was carried out on a collection of 207 clinical strains obtained from patients with primary *K. pneumoniae* infections. The *pks* colibactin gene cluster markers *clbB* and *clbN* [41] were jointly detected in 53 (25.6%) of the 207 *K. pneumoniae* strains. PCR detection of two additional colibactin genes *clbA* and *clbQ* yielded results concordant with those for *clbB* and *clbN*. Approximately 66% (35/53) of the colibactin-positive *K. pneumoniae* were the K1 type (Table 1). Statistical analysis revealed a significant correlation between the carriage of colibactin genes and K1 type (odds ratio, 19.4; 95% confidence interval, 8.8–42.9; *p*<0.0001). However, the presence of colibactin genes appeared to be irrelevant to the type of infection, as the colibactin-positive *K. pneumoniae* distributed evenly among isolates collected from cases of KLA (8/35; 22%), non-hepatic abscess (17/59; 28%), and non-abscess infections (28/113; 25%). The overall colibactin-positive rate was 25.6% (53/207). This was significantly higher than that reported by *Putze et al*, in which only five out of 141 (3.5%) *K. pneumoniae* strains isolated from Europe were colibactin-positive [27]. Our result indicates a close relatedness of the colibactin gene cluster to K1 type. The relatively higher prevalence of colibactin genes demonstrated here might be related to the fact that serotype K1 was the most frequently identified type in Taiwan [42], but it ranked eighth in European survey [43].

**Table 1:** Factors associated with colibactin-positive *K. pneumoniae* isolates in Taiwan.

	K type^b^		Clinical characteristics		
	K1 (n = 49)	K2 (n = 35)	Pyogenic infection (n = 94)		Non-abscess^e^ (n = 113)
			KLA^c^ (n = 35)	Non-hepatic abscess^d^ (n = 59)	
**colibactin (n = 53)** a	35 (71)	9 (26)	8 (22)	17 (28)	28 (25)
**OR (95% CI)**	19.4 (8.8–42.9)	1.0 (0.5–2.0)	0.8 (0.4–2.0)	1.3 (0.6–2.5)	0.9 (0.5–1.7)
***p*** **-value**	<0.0001	1	0.8325	0.5969	0.8731

Data are no. (%) of isolates. Statistical comparisons by Fisher's exact test are between the *pks*-positive group and the *pks*-negative group.

a *K. pneumoniae* strains in which the *clbB* and *clbN* were simultaneously detected by PCR with gene-specific primers are considered colibactin-positive.

b K1 and K2 capsular antigens were determined by PCR detection of the K-serotype-specific *wzx* locus with specific primers.

c *K. pneumoniae* strains from tissue-invasive cases that presented with the formation of liver abscesses were regarded as KLA isolates.

d *K. pneumoniae* strains from cases associated with abscesses at non-hepatic sites, including lesions that occurred as empyema, endophthalmitis, necrotizing fasciitis, septic arthritis, along with lung, epidural, parotid, paraspinal, splenic, renal, prostate, muscle, and deep neck abscesses.

e *K. pneumoniae* strains from non-abscess-related cases, including pneumonia without abscess, primary peritonitis, cellulitis, biliary tract infection, primary bacteremia with no original infectious foci identifiable, and catheter-related infections.

Chronic inflammation has been recognized as a risk factor for CRC and other cancers. In a colitis-associated CRC mouse model, the proliferation of a genotoxic *E. coli* commensal strain, NC101, was demonstrated to be favored by an inflammatory milieu and thus promoted tumorigenesis of CRC [26]. Several clinical studies have demonstrated the relationship between CRC, PLA and *K. pneumoniae* in Taiwan [17], [18], [20]. The hazard ratio of CRC was 3.36 times greater for patients with PLA as reported in a nationwide population-based 5-year follow-up study comprised of 274 PLA patients and 1,370 randomly selected subjects [18]. In another nationwide cohort study in Taiwan, of which 1257 PLA patients without prior cancers were followed-up from 2008–2012, it was reported that the incidence of CRC in PLA patients was significantly raised as compared to the control group [17]. In addition, a retrospective study of PLA patients at a medical center in Taiwan has further indicated that the risk of CRC was 2.68 times greater for patients with *K. pneumoniae*-caused PLA than those with non-*K. pneumoniae* PLA [20]. Our discovery on the high prevalence of colibactin-positive *K. pneumoniae* in Taiwan, predominating among K1 strains, therefore poses an intriguing opportunity in studying the link between genotoxic *K. pneumoniae* and CRC. Whether the CRC-associated KLA strains correlate to the presence of colibactin genes remains to be elucidated. However, as we report here that 25–28% of *K. pneumoniae* isolates from non-KLA infections were also colibactin-positive, the possibility that other types of *K. pneumoniae* infections may contribute to the development of CRC and/or other types of cancers cannot be ignored. The role of genotoxic *K. pneumoniae* in tumorigenesis deserves further studies.

## Conclusions

Here we report the complete genetic structure of KPHPI208 analyzed by comparative genomics approaches. The 208-kb genomic island contains eight genomic modules (GM1-GM8), including a *pks* colibactin gene cluster which was first identified and characterized in *E. coli* IHE3034 [24]. The *pks* colibactin gene cluster within the GM1 of KPHPI208 shares a striking ∼100% sequence identity with the *E. coli* version, suggesting that their functions and regulation are conserved. Accumulating evidence has pointed out the role of colibactin-producing *E. coli* in the development of chronic inflammation and CRC in humans [22], [24], [26]. In Taiwan, it has also been reported that *K. pneumoniae*-caused PLA significantly increased the risk of subsequent CRC [17], [18], [20]. The carriage of *pks* colibactin gene cluster may thus serve as a molecular basis underlying the epidemiological link between *K. pneumoniae* and CRC. Indeed, transient infection of BNL cells with *K. pneumoniae* 1084 induced DSBs, as revealed by the formation and accumulation of γH2AX foci. This genotoxic capacity of *K. pneumoniae* 1084 required the production of colibactin because it was abolished by the deletion of *clbA* gene and was restored to the wild type level by the complementation with a *clbA* coding plasmid. Moreover, in our KLA mouse model, *K. pneumoniae* 1084 exhibited an enhanced ability to induce DNA damage in the liver parenchymal cells when compared with the isogenic *clbA* deletion mutant. Furthermore, more than 25% of *K. pneumoniae* strains isolated from Taiwan carried the colibactin genes, and two-third of them (66%) belonged to the K1 type which is responsible for the development of invasive disease, such as PLA. The discovery of genotoxic *K. pneumoniae* as well as the determination of its complete genetic environment will help to elucidate the link between *K. pneumoniae*, PLA, and CRC.
